# Connections between metabolism and epigenetics: mechanisms and novel anti-cancer strategy

**DOI:** 10.3389/fphar.2022.935536

**Published:** 2022-07-22

**Authors:** Chen Chen, Zehua Wang, Yanru Qin

**Affiliations:** Department of Oncology, The First Affiliated Hospital of Zhengzhou University, Zhengzhou, China

**Keywords:** cancer metabolism, epigenetics, immunity, novel anti-cancer strategy, oncology

## Abstract

Cancer cells undergo metabolic adaptations to sustain their growth and proliferation under several stress conditions thereby displaying metabolic plasticity. Epigenetic modification is known to occur at the DNA, histone, and RNA level, which can alter chromatin state. For almost a century, our focus in cancer biology is dominated by oncogenic mutations. Until recently, the connection between metabolism and epigenetics in a reciprocal manner was spotlighted. Explicitly, several metabolites serve as substrates and co-factors of epigenetic enzymes to carry out post-translational modifications of DNA and histone. Genetic mutations in metabolic enzymes facilitate the production of oncometabolites that ultimately impact epigenetics. Numerous evidences also indicate epigenome is sensitive to cancer metabolism. Conversely, epigenetic dysfunction is certified to alter metabolic enzymes leading to tumorigenesis. Further, the bidirectional relationship between epigenetics and metabolism can impact directly and indirectly on immune microenvironment, which might create a new avenue for drug discovery. Here we summarize the effects of metabolism reprogramming on epigenetic modification, and vice versa; and the latest advances in targeting metabolism-epigenetic crosstalk. We also discuss the principles linking cancer metabolism, epigenetics and immunity, and seek optimal immunotherapy-based combinations.

## 1 Introduction

**FIGURE 1:**
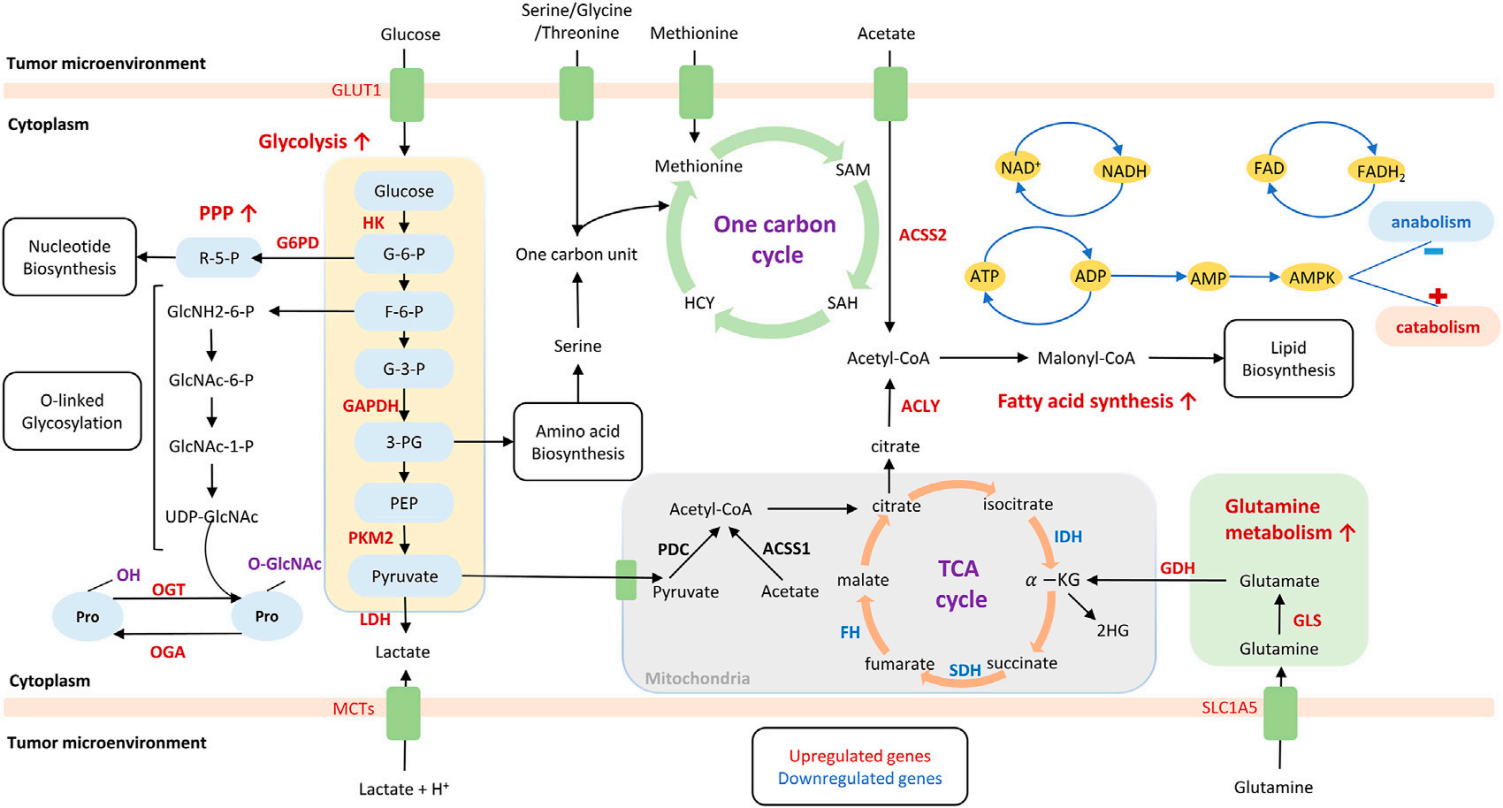
Metabolism reprogramming in cancer cells. Metabolism reprogramming is characterized by a class of altered pathway, including enhanced glycolysis with increased lactate production, and enhanced pentose phosphate pathway, fatty acid synthesis, and glutamine metabolism. These metabolic pathways support energy supply and macromolecule biosynthesis, such as nucleotides, amino acids, and lipids. Metabolites that are produced by altered metabolism have the potential to control signaling or epigenetic pathways by regulating reactive oxygen species, acetylation, and methylation. Upregulated genes or proteins are labels red, whereas downregulated genes or proteins are labeled blue. GLUT, glucose transporter; MCT, monocarboxylate transporter; SLC1A5, solute carrier family 1 member 5; TCA, Tricarboxylic acid cycle; G6PD, glucose-6-phosphate dehydrogenase; HK, hexokinase; GAPDH, glyceraldehyde-3-phosphate dehydrogenase; PKM, pyruvate kinase M 2; LDH, lactate dehydrogenase; ACSS2, Acyl-CoA short-chain synthetase-2; ACSS1: Acyl-CoA short-chain synthetase-1; ACLY: ATP citrate lyase; GLS, glutaminase; GDH, glutamate dehydrogenase; PDC: pyruvate dehydrogenase complex; FH, fumarate hydratase; SDH, succinate dehydrogenase; IDH1/2, isocitrate dehydrogenase 1/2; HCY, homocysteine; PPP, pentose phosphate pathway; ATP, adenosine triphosphate; ADP, adenosine diphosphate; AMP, adenosine monophosphate; AMPK, AMP-activated protein kinase; OGT, O-GlcNAc transferase; OGA, O-GlcNAcase.

Cancer metabolism is based on the principle that cancer cells undergo metabolic adaptations to sustain their uncontrolled proliferation. Such adaptations render malignant cells to exhibit altered metabolism compared to the normal cells. In 1920s, Warburg firstly proposed (Kaye, 1998; Chinnaiyan et al., 2012) that cancer cells display enhanced glycolysis and increased secretion of lactate even with abundant oxygen supply. This phenomenon is termed as “Warburg effect” or aerobic glycolysis. Moreover, an emerging class of metabolic alterations enables tumor cells to take up available ample nutrients and utilize them to produce ATP, generate biosynthetic precursors for cell anabolism, and tolerate stresses related to malignancy, such as hypoxia and nutrient starvation (Owen et al., 2002; Koppenol et al., 2011; Lunt and Vander Heiden, 2011; Metallo et al., 2011; Mullen et al., 2011; Wise et al., 2011; Cantor and Sabatini, 2012; Ahn and Metallo, 2015). In this context, cancer metabolism provides a selective advantage during tumorigenesis. Metabolic reprogramming (Figure 1) is now recognized as a hallmark of cancer (Hanahan and Weinberg, 2011; Pavlova and Thompson, 2016), which could be intrinsically regulated by genotype and epigenotype, or extrinsically affected by tumor microenvironment (TME).

Epigenetics was firstly established by Conrad Waddington in 1942 (Cairns et al., 2011), which refers to the study of modification in gene expression or cellular phenotype that occurs without changes in DNA nucleotide sequences (Possemato et al., 2011). The basic unit of chromatin organization is nucleosome, which is composed of DNA and histone octamer. Chromatin state is a dynamic event that controls gene transcription. Epigenetic modification of gene expression occurs at the DNA, histone, and RNA level. The most well-characterized examples are DNA methylation, histone methylation, acetylation, phosphorylation, ubiquitination, and microRNA-dependent gene silencing (Margueron and Reinberg, 2010). It is widely recognized that epigenetic dysfunction is a common feature of many cancers (Ribich et al., 2017). Numerous excellent reviews have summarized the biology fundamentals of chromatin-modified proteins (CMPs) (Tessarz and Kouzarides, 2014; Piunti and Shilatifard, 2016; Soshnev et al., 2016) and the therapeutic potentials to target CMPs in tumor (Pfister and Ashworth, 2017).

For almost a century, our focus in cancer is dominated by oncogenic mutations. Until recently, the connection between metabolism and epigenetics was emphasized in cancer biology. Metabolism reprogramming is known to affect epigenetic landscapes through different mechanisms. Conversely, epigenetic regulation contributes to altered metabolic activities. Hence, cancer metabolism and epigenetics are highly interwoven in a reciprocal manner. This great breakthrough has gained wide interest in targeting both altered metabolism and modified epigenetics. However, whether these two hallmarks synergistically attack tumor remains unknown. Noteworthy, such a complex relationship has the potential to affect immune system, such as trained immunity, T cell activation, macrophage activation. A novel strategy is to target epigenetics-metabolism axis in combination with immunotherapy, potentially boosting more potent antitumor responses.

In this review article, we firstly summarize the metabolic alterations that drive epigenetic changes in cancer, and vice versa. We next describe the therapeutic opportunities by targeting metabolism-epigenetic crosstalk. Further, we discuss the principles linking metabolism, epigenetics to immunity and introduce the rationale for novel immunotherapy-based combinations. Our aim is to introduce the fundamentals of connection between metabolism and epigenetics in cancer biology and discuss potential pharmacological strategies that can exploit the metabolism and epigenetics in malignancy.

## 2 Metabolism shapes the epigenetic state of cancer cells

Tumors are likely to harbor epigenetic changes driven by their cellular metabolism. There are several different mechanisms explaining the influx from metabolism to chromatin.

### 2.1 Metabolites are either substrates or co-factors for epigenetic enzymes

Epigenetic enzymes employ several metabolic intermediates as substrates or co-factors to carry out post-translational modifications of DNA and histone (Katada et al., 2012), which in turn influence metabolic gene expression. Examples of such metabolites include: SAM, α -KG, and FAD that participate in DNA and histone methylation; acetate, acetyl-CoA and NAD^+^ that mediate histone acetylation (Thakur and Chen, 2019). These key metabolites are produced in multiple pathways mediated by metabolic enzymes: SAM from one-carbon metabolism, α -KG and FAD^+^ from the TCA cycle, acetyl-CoA from glycolysis and glutamine metabolism, and NAD^+^ from the conjunction of glycolysis and oxidative phosphorylation (Wang and Lei, 2018). The fundamental interface between metabolism and epigenetics has been summarized in Table 1.

**TABLE 1:** Fundamental interface of metabolism and epigenetics.

Metabolism pathway	Metabolic enzyme	Metabolites	Epigenetic enzyme	Epigenetic regulation
One-carbon cycle	MAT	SAM/SAH	KMT, PRMT	DNA and histone methylation
TCA cycle	FADS	FAD/FADH2	LSD	Histone demethylation
TCA cycle	IDH, GLUD	α-KG	TET and JmjC demethylase	DNA and histone demethylation
TCA cycle	ACSS1, ACSS2, ACLY	Acetyl-CoA/CoA	HAT	Histone acetylation
Glycolysis/TCA cycle	NMNAT	NAD+/NADH	SIRT, PARP	Histone deacetylation
TCA cycle	NA	AMP/ATP	AMPK	Phospharylation
Hexosamine	NA	GlcNac	OGT	GlcNacylation

MAT, methionine adenosyltransferase; SAM, S-adenosylmethionine; SAH, S-adenosylhomocysteine; KMT, Lysine methyltransferase; PRMT, protein arginine methyltransferase; TCA, Tricarboxylic acid; ACSS, acetyl-CoA synthetase short-chain family member; ACLY, ATP citrate lyase; HAT, histone acetyltransferase; NMNAT, nicotinamide mononucleotide adenylytransferase; PARP, poly-ADP ribose polymerase; FADS, flavin adenine dinucleotides; LSD, lysine specific demethylase; IDH, isocitrate dehydrogenase; GLUD, glutamate dehydrogenase; TET, ten-eleven translocation methylcytosine dioxygenase; JmjC, Jumonji N/C-terminal domains; ATP, adenosine triphosphate; ADP, adenosine diphosphate; AMP, adenosine monophosphate; AMPK, AMP-activated protein kinase; GlcNac, O-linked N-acetylglucosamine; OGT, O-linked N-acetylglucosamine transferase; NA, Not Applicable

### 2.2 SAM/SAH ratio affects DNA and histone methylation

#### 2.2.1 SAM/SAH

DNA and histone methylation are respectively mediated by DNA methyltransferase (DNMT) enzymes and histone methyltransferase (HMT) enzymes (Varier and Timmers, 2011), both of which utilize S-Adenosyl-methionine (SAM) as a major methyl donor. Methylation is to transfer a methyl group from SAM to the receptor, and the remaining residue is S-adenosyl-homocysteine (SAH) that is inhibitory to methyltransferase. SAM is derived from one-carbon metabolism that plays integral roles in DNA synthesis and methylation reaction. The most studied metabolites, like glucose and glutamine, feed into the one-carbon cycle and increase the availability of SAM. Both global DNA hypomethylation and site-specific CpG hypermethylation are frequent epigenetic abnormities observed in cancer (Sandoval and Esteller, 2012), while histone methylation may activate or repress gene transcription (Vakoc et al., 2005; Berger, 2007; Bernstein et al., 2007). Therefore, SAM/SAH ratio directly affect the methylation status of chromatin.

### 2.3 TCA cycle metabolites regulate DNA and histone demethylation

#### 2.3.1 TCA cycle metabolites

Reversal of DNA and histone methylation is catalyzed by DNA and histone demethylase. Histone demethylation is regulated by two classes of enzymes: lysine-specific demethylase family (LSD1 and LSD2) (Fang et al., 2010) and JmjC-containing family, both of which are dependent on ferrous adenine dinucleotide (FAD). Also, JmjC family is ferrous ion-dependent oxygenase requiring α -KG for the enzymatic activation (Shi et al., 2005; Klose et al., 2006). Likewise, DNA demethylation is modulated by TET-family proteins (TET1, TET2, and TET3), which are also FAD- and α -KG-dependent dioxygenase (Bhutani et al., 2011; He et al., 2011; Ito et al., 2011). Both FAD and α -KG are intermediary metabolites produced in TCA cycle. Other TCA metabolites, such as succinate and fumarate, are identified as antagonists for JmjC-containing family demethylase (Xiao et al., 2012). Therefore, TCA cycle metabolites regulate epigenetic marks on DNA and histone.

### 2.4 Acetyl-CoA, NAD^+^ and acetate influence histone acetylation

#### 2.4.1 Acetyl-CoA

Histone acetylation is another important epigenetic modification that depends on histone acetyltransferase (HAT) and histone deacetylase (HDAC) (Shahbazian and Grunstein, 2007). Acetyl-CoA is a pivotal metabolite for energy production and anabolic process (Wellen and Thompson, 2012; Pietrocola et al., 2015). HAT transfers the acetyl moiety of acetyl-CoA to lysine residues of histone, while HDAC is responsible for removing the acetyl group to reverse histone acetylation. It is well-known histone acetylation can increase nucleosome mobility and activate transcription elongation (Racey and Byvoet, 1971; Cai et al., 2011). Previous study figured out, in yeast and mammalian cells, the glycolysis dynamically governs the acetyl-CoA quantity and correspondingly regulates HAT-dependent histone acetylation (Friis et al., 2009; Cai et al., 2011; Lee et al., 2014).

#### 2.4.2 NAD^+^

Histone deacetylation is catalyzed by two kinds of deacetylases: zinc-dependent and NAD^+^-dependent proteins. Deacetylation results in the tight wrapping of DNA by histone and hence promotes gene repression and silence (Imai et al., 2000; Finkel et al., 2009). Similarly, some metabolites function as antagonists that inhibit the activities of HDAC. For example, butyrate can robustly antagonize HDACs I, II and IV (Candido et al., 1978). Also, NAD^+^ is regarded as a catalytic co-factor for HDAC III to mediate histone deacetylation (Thakur and Chen, 2019). Further, evidence illustrated higher histone deacetylation levels are associated with poorer prognosis (Kurdistani, 2011).

#### 2.4.3 Acetate

Acetate has been implicated in driving histone acetylation and deacetylation. Recently, the role of acetate in the interaction between metabolism and epigenetics has been emphasized during tumorigenesis. Under hypoxia, cancer cells decrease the reliance on glucose and glutamate and inversely increase the demand of acetate as a substitute carbon source for lipid synthesis (Kamphorst et al., 2014). Consequently, acetate must be converted to acetyl-CoA either by ACSS1 in mitochondria or by ACSS2 in the cytoplasm or nucleus (Figure 1). There is already evidence that both acetate and acetyl-CoA facilitate tumor growth by histone acetylation in yeast (Cai et al., 2011). ACSS2, as the only known enzyme utilizing free acetate in nucleus (Moffett et al., 2020), could shape the epigenetic landscape via selective histone acetylation. More specifically, ACSS2 is translocated from cytoplasm to the nucleus supplying a local of acetyl-CoA (Chen et al., 2017), which contributes to all kinds of acetylation reactions in cell nuclei. One study indicated (Gao et al., 2016), under hypoxia condition, ACSS2 catalyzes the conversion of acetate to acetyl-CoA in the hepatoma carcinoma cells, facilitating the hyper-acetylation of histone K3K9, H3K27, and H3K56 and thereby upregulating the expression of lipogenic enzymes. This explains how acetate links metabolite levels to epigenetic regulation and gene transcription. Otherwise, ACSS2 acts to recycle acetate generated from HDAC-mediated deacetylation reactions under metabolic stresses, replenishing the cytoplasmic and nuclear storage and thus supporting chromatin remodeling events (Moffett et al., 2020).

### 2.5 ATP/AMP ratio controls histone phosphorylation

#### 2.5.1 ATP/AMP

Some kinase could be translocated to nucleus and straightly phosphorylate histone (Baek, 2011). For example, AMP-activated protein kinase (AMPK) acts as sensory signal of ATP/AMP ratio (Hardie, 2011). Conversion of ATP to AMP aids in anabolic process via AMPK-mediated pathway, whereas catabolism relies on the opposite switch from AMP to ATP. Owing to metabolic stress and low ATP/AMP ratio, AMPK is activated to phosphorylate histone H2B on serine 36 that triggers gene expression in favor of tumor survival (Bungard et al., 2010).

### 2.6 Hexosamine biosynthetic pathway mediates protein glycosylation

#### 2.6.1 O-GlcNAc

Protein glycosylation is carried by opposite actions of O-GlcNAc transferase (OGT) and O-GlcNAcase (OGA), respectively responsible for the addition and removal of O-GlcNAc from proteins. One of the most common features that cancer cells demonstrate is OGT overexpression leading to protein hyper-glycosylation (Pinho and Reis, 2015). Typically, O-GlcNAc is produced in Hexosamine biosynthetic pathway (HBP). In this pathway, glucose is firstly converted into glucose-6-P and then fructose-6-P. A series of metabolites, such as acetyl-CoA, UTP, glutamine, subsequently participate in the production of UDP-GlcNAc, the activated substrate for O-GlcNAcylation. Therefore, HBP integrated various metabolism pathways. Upregulation of HBP is associated with abnormal O-GlcNAcylation and more invasive behavior (Caldwell et al., 2010; Wellen et al., 2010; Itkonen et al., 2013; Onodera et al., 2014; Lucena et al., 2016). Recently, studies confirm that enhanced glycolysis aids in protein glycosylation (Wong et al., 2017). Moreover, OGT is associated with TETs to control O-GlcNAcylation of histone H2B for activation of gene transcription (Chen et al., 2013; Ito et al., 2014), while OGT is coordinated with EZH2 to modulate H3K27me3 for silence of tumor suppressor genes (Chu et al., 2014).

**FIGURE 2:**
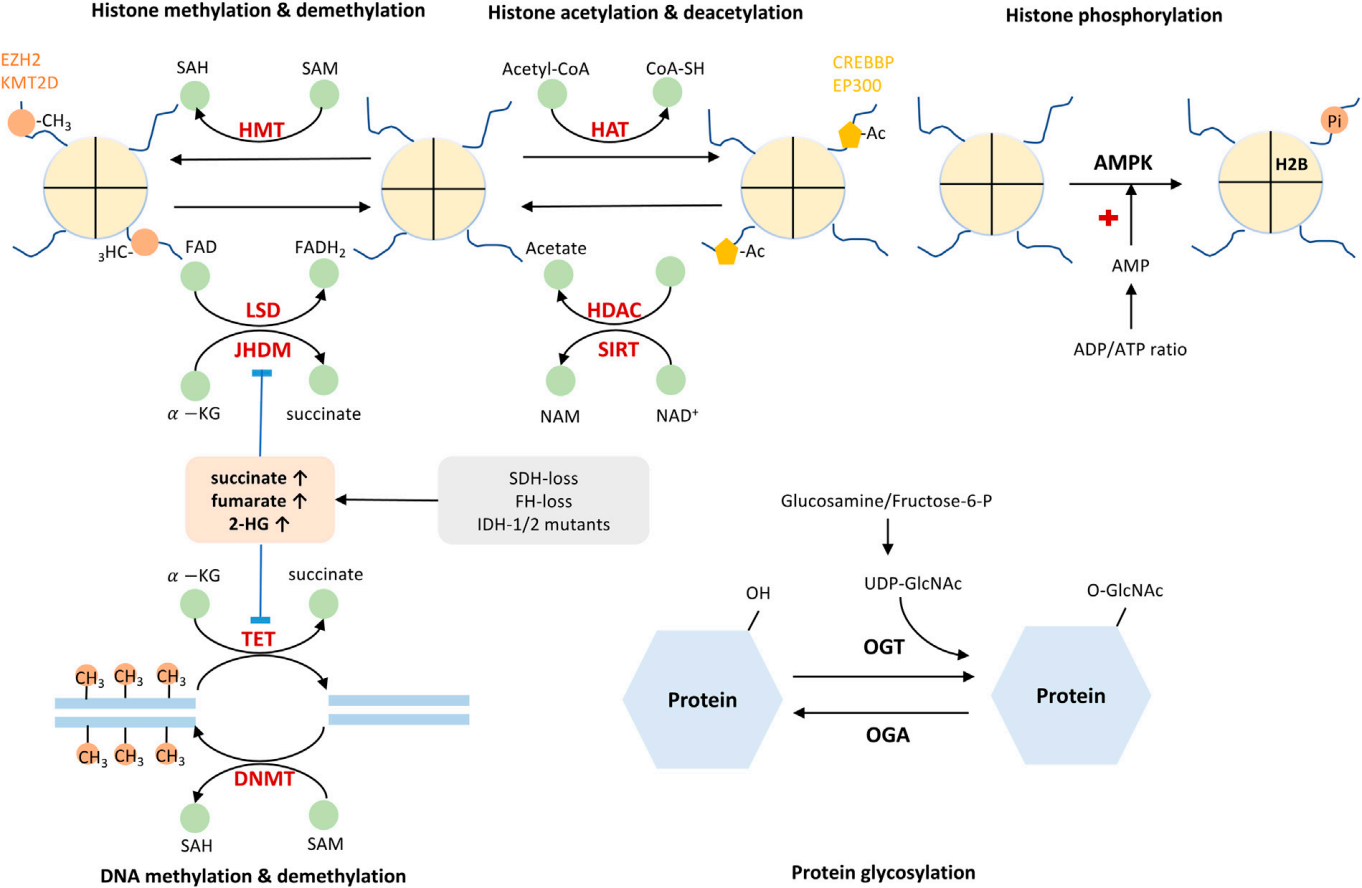
Cellular metabolites serve as co-factors or substrates for epigenetic enzymes. Addition or removal of epigenetic marks is catalyzed by epigenetic enzymes, of which process relies on several critical metabolites. SAH/SAM, NAD^+^/NADH, Acetyl-CoA/Co-A, ATP/ADP ratio act as important molecules or signals governing epigenetic modifications. In addition, Metabolites such as succinate, fumarate, 2-HG, and lactate could inhibit the activity of epigenetic enzymes. HMT, histone methyltransferase; LSD, lysine-specific histone demethylase; JHDM, Jumonji domain-containing histone demethylase; HAT, histone acetyltransferase; HDAC, histone deacetylase; SIRT, sirtuins; DNMT, DNA methyltransferase; TET, ten-eleven translocation methylcytosine dioxygenase; SAM, S-adenosylmethionine; SAH, S-adenosylhomocysteine;  α− KG,    α -ketoglutarate; NAM, nicotinamide; NAD^+^, nicotinamide adenine dinucleotide (oxidized); FAD, flavin adenine dinucleotide (oxidized); FADH_2_, flavin adenine dinucleotide (reduced); FH, fumarate hydratase; SDH, succinate dehydrogenase; IDH1/2, isocitrate dehydrogenase 1/2; EZH2, enhancer of zeste 2 polycomb repressive complex 2 subunit; KMT2D, histone-lysine N-methyltransferase 2D. AMPK, AMP-activated protein kinase; Pi, phosphate group; OGT, O-GlcNAc transferase; OGA, O-GlcNAcase.

Taken together, either methylation or acetylation controls the activation and repression of gene transcription. This event is balanced by various epigenetic enzymes. The cellular metabolites, such as SAM/SAH, acetyl-CoA/CoA, NAD^+^/NADH, ATP/AMP ratio, commonly act as substrate or co-factors for these epigenetic-based enzymes (Table 2, Figure 2). Their fluctuating concentrations could regulate the epigenetic profile and affect gene transcription.

**TABLE 2:** Metabolites are either substrates or co-factors for epigenetic enzymes in cancer biology.

Epigenetic enzymes	Examples	Substrates or Co-factors	Mechanisms
DNA methylation and demethylation			
DNA methyltransferase	DNMTs	SAM/SAH (methionine cycle)	Methyl donors for methyltransferases
DNA demethylase	TETs	α-KG, 2HG, succinate, fumarate, vitamin C, FAD/FADH_2_	Co-factors for α-KG-utilizing dioxygenases; Inhibition of α-KG-utilizing dioxygenases
Histone acetylation and deacetylation			
Histone acetyltransferase	HATs	Acetyl-CoA (TCA cycle/acetate)	Acetyl donors for acetyltransferases
Histone deacetylases	HDAC, SIRT	NAD^+^, nicotinamide, β-Hydroxybutyrate, succinyl-CoA, butyrate	Activation or inhibition of histone deacetylase; Histone succinylation
Histone methylation and demethylation			
Histone methyltransferase	Lysine: PKMTs, Arginine: PRMTs	SAM/SAH (methionine cycle)	Methyl donors for methyltransferases
Histone demethylases	KDMs: LSD, JmjC	α-KG, 2HG, succinate, fumarate, vitamin C, FADH_2_	Co-factors for α-KG-utilizing dioxygenases; Positive regulators of LSD; Inhibition of α-KG-utilizing dioxygenases
Histone phosphorylation			
Histone kinase	AMPK	ATP/AMP	Phosphate donors for protein kinase
Protein glycosylation			
Protein glycosylase	OGT, OGA	O-GlcNAc	O-GlcNAc donors for protein glycosylation

### 2.7 Genetic mutations of metabolic enzyme that modify epigenome

Mutations in metabolic enzymes subject the cells to tumorigenesis. Such changes facilitate the accumulation of metabolites that ultimately lead to epigenetic dysfunction (DeBerardinis and Chandel, 2016) and immunosuppression (Table 3).

**TABLE 3:** The effect of oncometabolites on epigenetic dysfunction and immunosuppression.

Oncometabolite	Metabolic enzymes	Epigenetic dysfunction	Immunosuppressive effect	Malignancies	References
D-2-hydroxyglutarate	IDH1/2	DNA and histone hypermethylation	NA	Glioblastoma multiforme, ALL, Chondrosarcoma, Cholangiocarcinoma	Dang et al. (2009); Amary et al. (2011); Borger et al. (2014); Shim et al. (2014); Waterfall et al. (2014); Colvin et al. (2016)
L-2-hydroxyglutarate	L2HGDH	DNA and histone hypermethylation	NA	Brain tumors, Renal cell carcinoma	Aghili et al. (2009); Rogers et al. (2010)
Succinate	SDH	DNA and histone hypermethylation	TAM marker gene expression ↑	Pheochromocytomas, Paragangliomas	Hao et al. (2009); Bardella et al. (2011); Zhang et al. (2011); Yang et al. (2013); Williamson et al. (2015); Jiang and Yan, (2017); Mu et al. (2017)
			IL-6 secretion ↑		
Fumarate	FH	DNA and histone hypermethylation	Neutrophils, T-cell, B-cell response ↓	Pheochromocytomas, Paragangliomas	Kinch et al. (2011); Fieuw et al. (2012); Sullivan et al. (2013); Zheng et al. (2013b); Castro-Vega et al. (2014); Shanmugasundaram et al. (2014); Yang et al. (2014); Jin et al. (2015); Zheng et al. (2015)
			Inhibiting DC maturation		
			CD150, CD40, CD86 expression ↓		
			CTLA-4, PD-L1 expression ↑		
			IL-6, IL-1β, TNF-α secretion ↓		
Lactate	MCT/LDH	Histone acetylation	PD-1, PD-L1, CTLA-4 expression ↑	Lung carcinoma, Melanoma, Prostate cancer	(Colegio et al., 2014; El-Kenawi et al., 2019)
			Inhibiting the differentiation of monocytes to DCs		
			Inhibiting the differentiation of progenitor cells to CD4^+^ and CD8^+^ T-cell		

IDH1/2, isocitrate dehydrogenase; L2HGDH, L-2-hydroxyglutarate dehydrogenase; SDH, succinate dehydrogenase; FH, fumarate hydratase; MCT, monocarboxylate transporter; LDH, lactate dehydrogenase; TAM, tumor-associated macrophages; ALL, acute lymphoblastic leukemia; NA, not applicable.

One example is to generate oncometabolite. Oncometabolite refers to metabolites whose great quantity increases markedly in tumors compared with normal cells (Nowicki and Gottlieb, 2015). This new term is used to describe metabolites for which 1) there is a well-characterized mechanism connecting mutations in metabolic enzymes to accumulation of a certain metabolite; 2) there is convincing evidence for some metabolites as a predisposition to tumorigenesis. Oncometabolites are frequently associated with aberrant DNA damage and enable the tumor microenvironment (TME) more invasive. Currently, D-2-hydroxyglutarate (D2HG), L-2-hydroxyglutarate (L2HG), succinate, fumarate, and lactate are recognized oncometabolites.

#### 2.7.1 D2HG and L2HG

The first emphasized oncometabolite is D2HG, a reduced form of the TCA cycle intermediate α -ketoglutarate, which is scarce in normal tissues but rises to a higher concentration in tumors (Xu et al., 2011). This oncometabolite is caused by NADP^+^-dependent isocitrate dehydrogenase (IDH1 or IDH2) mutation. High levels of D2HG inhibit the activity of TET-family DNA and JmjC family histone demethylase. Overall, cancer cells harboring IDH1/IDH2 mutations display hypermethylation of DNA and histone (Figueroa et al., 2010; Losman et al., 2013). Mutant-IDH1/IDH2 and their relationship to D2HG have been reviewed extensively elsewhere (Losman and Kaelin, 2013). These mutations frequently occur in gliomas, blood cancer, glioblastoma multiforme, and cholangiocarcinoma (Yan et al., 2009; Vatrinet et al., 2017). Another reduced form of α -ketoglutarate is L2HG that is accumulated due to loss-of-function mutations of L-2-hydroxyglutarate dehydrogenase (L2HGDH) (Aghili et al., 2009; Rogers et al., 2010). The increased levels of L2HG have been observed in renal cell carcinoma and brain tumors (Shim et al., 2014).

#### 2.7.2 Succinate and fumarate

This principle also applies to another two oncometabolites: succinate and fumarate (Yang et al., 2013). Mutational inactivation of succinate dehydrase (SDH) and fumarate hydratase (FH) respectively contributes to the stacking up of succinate and fumarate (Baysal et al., 2000; Tomlinson et al., 2002; Gottlieb and Tomlinson, 2005), both of which interfere with α KG-dependent dioxygenases, namely DNA and histone demethylase (Nowicki and Gottlieb, 2015). Consequently, deficiency of SDH and FH activity results in DNA and histone hypermethylation, supporting the notion that oncometabolites are potent modifiers of the epigenome. Other studies provided additional layers of metabolic control of epigenome. FH is observed to be O-GlycNAcylated and consequently bring changes in histone methylation (Wang et al., 2017). Another research proposed that the enrichment of fumarate facilitates epithelial-to-mesenchymal-transition (EMT) through inhibiting TET methylase (Sciacovelli et al., 2016). Therefore, oncometabolites perform their biological functions outside of conventional pathways and play quantitative roles leading to aberrant epigenome. Additionally, emerging evidence supports that both succinate and fumarate contribute to immunosuppressive polarization and T cell exhaustion, thereby making the tumor microenvironment more suitable for cell migration. Explicitly, succinate can upregulate tumor-associated macrophages (TAM) marker gene expression, such as Arg1, Fizz1, Mhl1, and Mgl2. The expression of succinate receptor 1 is also associated with immune inhibitory proteins, such as PD-L1, PD-1, and CTLA-4. Moreover, fumarate could downregulate neutrophils, T-cell, and B-cell responses, inhibit dendritic cell (DC) maturation, and motivate CTLA-4 and PD-L1 expression.

#### 2.7.3 Lactate

To ensure adequate ATP supply, the malignant transformation is associated with an upregulated glycolysis (de Groof et al., 2009). Cancer cells upregulate glycolytic enzymes and metabolic transporters, which is connected with lactate overproduction. A new discovery considered lactate might have an effect on lysine residues of histone, acting in a similar way to acetylation and gene activation (Hou et al., 2019; Zhang et al., 2019). This phenomenon is based on the conversion of lactate to acetyl residues and thereby stimulates tumor angiogenesis. The accumulation of lactate also exerts an immunosuppressive effect on TME through inhibiting the differentiation and maturation of DC and T cell (Gottfried et al., 2006).

#### 2.7.4 PHGDH, PRODH, and NNMT

Cancer-specific mutations of metabolic enzymes with implications in epigenetic regulation have been reported. Phosphoglycerate dehydrogenase (PHGDH) is overexpressed in breast cancer and melanoma (Locasale et al., 2011; Possemato et al., 2011), directing the metabolism toward the serine biosynthesis pathway. Serine provides methyl donors to one-carbon metabolism, thereby affecting cellular epigenetics (Locasale, 2013). Conversely, PHGDH silence can downregulate serine synthesis leading to tumor growth suppression (Locasale et al., 2011; Possemato et al., 2011). Another example is proline dehydrogenase (PRODH) that catalyzes proline to produce pyrroline-5-carbonxylate (P5C), which is sequentially converted into glutamate and α -KG to affect epigenome (Phang et al., 2013). Studies showed amplification of PRODH in immunodeficient mice displayed tumor-suppressive characters (Liu et al., 2010). Nicotinamide N-methyltransferase (NNMT) also modulates epigenetic events in cancer cells. NNMT catalyzes the transfer of methyl group from SAM to nicotinamide. Overexpression of NNMT hampers SAM-dependent methylation of DNA and histone, along with the procurement of more invasive phenotype (Ulanovskaya et al., 2013).

As summarized, mutations in genes encoding metabolic enzymes have been recognized in caner, but they are rare. These lesions in genes related to metabolism constitute a new class of cancer-associated mutations that is able to subvert normal epigenetic regulation. It is tempting to speculate that these mutations provide the hope of identifying novel targets.

## 3 Epigenetic events contribute to altered metabolism in cancer

### 3.1 DNA methylation

A number of metabolic enzymes are altered attributing to DNA methylation. Examples of such enzymes involve Fructose-1,6-bisphosphastase (FBP-1), fructose-1,6-bisphosphatase (FBP-2), glucose transporter 1 (GLUT-1), Hexokinase (HK2), and pyruvate kinase isozyme 2 (PKM-2).

As reported, promoter hypermethylation leads to the silence of FBP-1 and FBP-2 in gastric, colon, liver, and breast cancers (Kamphorst et al., 2014; Gao et al., 2016). Both FBP-1 and FBP-2 are rate-limiting enzymes for gluconeogenesis that antagonize glycolysis. Theoretically, the silence of FBP-1 or FBP-2 contributes to glycolytic phenotype, supporting macromolecular biosynthesis and energy production. DNA methylation also mediates the gene overexpression of GLUT-1 that transports glucose from tumor microenvironment to cytoplasm (Lopez-Serra et al., 2014). Oppositely, promoter hypomethylation results in the upregulation of HK2 in glioblastoma and hepatic carcinoma (Chen et al., 2011; Wolf et al., 2011) and the overexpression of PKM2 in multiple cancer types (Desai et al., 2014).

In brief, increased HK2 and PKM-2 levels promote enhanced glycolysis, while the silence of FBP-1 and FBP-2 limit gluconeogenesis. DNA methylation contributes to a higher glycolytic influx, which is beneficial to the proliferation of tumor cells.

### 3.2 Histone modifications

Sirtuins (SIRTs), an enzyme catalyzing histone deacetylation, has been shown to function in cancer metabolism. Examples of epigenetic enzymes are SIRT6, SIRT7, and SIRT2.

#### 3.2.1 SIRT6

NAD^+^-dependent SIRT6 optimizes energy homeostasis by regulating histone acetylation (Xiao et al., 2010). SIRT6 could directly repress glycolysis in the HIF1 α -dependent way, thereby it acts as a tumor suppressor by inhibiting the Warburg effect (Zhong et al., 2010; Sebastián et al., 2012). Instead, SIRT6 knockdown shifts the cell metabolism towards a “glycolytic phenotype” inducing malignancy aggressiveness. Specific deletions in SIRT6 have been observed in colon, pancreatic, and hepatocellular cells (Zhang and Qin, 2014). Also, a growing body of evidence demonstrates that SIRT6 upregulates hepatic gluconeogenic gene expression and increases glycerol release from adipose tissue. These findings underline the potential to target SIRT6 for modulating cancer metabolism (Roichman et al., 2021).

#### 3.2.2 SIRT7

SIRT7 could directly interacts with MYC that mediates the transcription of almost all the genes involved in glycolysis and glutaminolysis (Barber et al., 2012; Shin et al., 2013). SIRT7 selectively catalyzes H3K18 deacetylation that is a repressive mark (Wong et al., 2017). Hence, SIRT7 plays an opposite role in MYC-mediated metabolic reprogramming.

#### 3.2.3 SIRT2

Compared to SIRT6/7, SIRT2 promotes cancer metabolism through stabilizing MYC (Liu et al., 2013). SIRT2 specifically deacetylases H4K16, resulting in decreased expression of ubiquitin-protein ligase NEDD4. NEDD4 serves as a negative regulator of MYC through ubiquitination and degradation (Wong et al., 2017). Consequently, SIRT2 facilitates MYC-dependent transcription and oncogenesis.

## 4 Novel cancer therapy targeting metabolism-epigenetic crosstalk

### 4.1 Novel targets for cancer metabolism

Targeting metabolic enzymes might be novel strategy for cancer therapy. LDH-A, a metabolic enzyme responsible for the conversion of pyruvate to lactate, was recognized as the first metabolic target of the oncogene MYC (Shim et al., 1997). Appealing evidence manifested genetic or pharmacologic ablation of LDH-A is able to dwindle MYC-driven tumors in the xenograft models (Fantin et al., 2006; Le et al., 2010). Inhibition of LDH-A could delay the progression of myeloid leukemia (Wang et al., 2014) and diminish NSCLC without systemic toxicity in genetically engineered mouse models (Xie et al., 2014). Hence, LDH-A is a promising target in MYC-mutant tumors. Another attractive target is the glycolytic protein Hexokinase (HK2). Many tumors express high levels of HK2. Specific inhibition of HK2 delays tumor progression in mouse models of NSCLC and breast cancer (Patra et al., 2013). Targeting HK2 might be efficacious in highly glycolytic tumors. Besides, PHGDH, an enzyme that functions in the *de novo* serine synthesis, is found to overexpress in human melanoma and breast cancers (Locasale et al., 2011; Possemato et al., 2011). Targeting PHGDH in the one-carbon metabolism has been shown to delay tumor progression, though more studies are needed to confirm it. Additionally, the concept of oncometabolite opened a new window for targeted therapy. Small molecules targeting IDH1/IDH2 demonstrate positive outcomes in ongoing clinical trials (Yen et al., 2017). Taken together, targeting metabolic enzyme holds great promise in the treatment of malignancy (Olivares et al., 2015).

Targeting metabolism pathways, such as glycolysis, glutamine metabolism, mitochondrial metabolism, and autophagy, provides new opportunities for drug discovery scheme. In the certain context, metabolites produced from these metabolic pathways are able to affect epigenome. For example, metformin, an anti-diabetic drug, has been spotlighted on mitochondrial-mediated metabolic activity emerging as a key target for cancer therapy (Weinberg and Chandel, 2015). Because diabetic patients treated with metformin not only control their blood glucose level but also improve survival rate if cancer was diagnosed already (Evans et al., 2005). Biguanide phenformin also displayed anti-tumor effect by inhibiting mitochondrial complex I (Birsoy et al., 2014). Another example is BPTES [bis-2-(5-phenylacetamido-1, 2, 4-thiadiazol-2-yl) ethyl sulfide], one inhibitor of glutaminase activity, is being explored for anti-cancer characteristics (Xiang et al., 2015). Autography offers amino acids that fuel TCA cycle. Autography inhibition is confirmed to decrease tumor progression without significant toxicity in the mouse models of NSCLC and pancreatic cancers (Son et al., 2013; Karsli-Uzunbas et al., 2014). An alternative approach is to target acetate metabolism. As discussed above, mitochondria conventionally provide acetyl-CoA to the normal cells, whereas cancer cells also utilize acetate to support cell survival under hypoxia or nutrient deprivation (Schug et al., 2015). ACCS2, a cytosolic enzyme that converts acetate to acetyl-CoA, is dispensable for acetate metabolism and holds great promise for cancer therapy. In models of hepatocellular carcinoma, genetic loss of ACSS2 is likely to reduce tumor burden (Comerford et al., 2014). Human glioblastoma is sensitive to inhibitors of ACSS2 as well (Mashimo et al., 2014).

### 4.2 Reversal of epigenetic dysfunction by targeting metabolism

Over the past decades, a few studies represent how advances of metabolic effects on epigenetics can be translated into potential therapies. One strategy is to reverse epigenetic dysfunction by targeting cancer metabolism (Table 4).

**TABLE 4:** Reversal of epigenetic dysfunction by targeting metabolism.

Target pathway	Metabolic enzyme	Pharmacological agents	Mechanism	Indications	References
Glycolysis	Hexokinases	2-DG (phase-I/II)	2-DG suppresses hexokinase that is a rate-limiting enzyme for glycolysis; 2-DG reduces acetyl-CoA level, which inhibits the acetylation of histones in various cancer cell lines	lung cancer, breast cancer, pancreatic cancer, prostate cancer, lymphoma	Chen and Guéron, (1992); Liu et al. (2015)
Glutaminolysis	Glutaminase (GLS)	CB-839 (phase-I); Compound-968; Zaprinast	GLS inhibitors reduce acetyl-CoA and 2-HG level; Compound-968 decreases histone H3K4me3 in breast cancer and Zaprinast reduces H3K9me3 in IDH1-mutant cancer cells	AML, ALL, MM, NHL, pancreatic carcinoma	Robinson et al. (2007); Wang et al. (2010a); Simpson et al. (2012a); Simpson et al. (2012b); Elhammali et al. (2014)
Serine/glycine metabolism	PHGDH	shRNA to PHGDH	Inhibiting the process of *de novo* serine synthesis	NA	Locasale et al. (2011); Possemato et al. (2011)
One-carbon cycle	SAH hydrolase	DZNep; Adenosine Dialdehyde	Both agents could increase the SAH/SAM ratio and decrease DNA and histone methylation	NA	Jiang et al. (2008); Miranda et al. (2009); Momparler et al. (2012); Schäfer and Balleyguier, (2013); Momparler and Côté, (2015)
IDH1 inhibitor	IDH1-mutant	AG-120, IDH305, AG-881, BAY1436032, FT-2102, AGI-5198, GSK-321	IDH1 inhibitors suppress the production of 2-HG that is a kind of oncometabolite in IDH1-mutant cells; AGI-5198 prompts demethylation of H3K9me3 and H3K27me3 in IDH1-mutant chondrosarcoma cells; GSK-321 induces DNA hypomethylation in IDH1-mutant AML cells	AML, solid tumors, gliomas, hematologic malignancies	Rohle et al. (2013); Zheng et al. (2013a); Davis et al. (2014); Deng et al. (2015); Kim et al. (2015); Li et al. (2015); Okoye-Okafor et al. (2015)
IDH2 inhibitor	IDH2-mutant	AG-221, AG-881, AGI-6780	IDH2 inhibitors suppress the production of 2-HG that is a kind of oncometabolite in IDH2-mutant cells; AG-221 and AGI-6780 prompt demethylation of DNA and histone in IDH2-mutant cancer cells	AML, solid tumors, gliomas, hematologic malignancies	Wang et al. (2013); Kernytsky et al. (2015)
NNMT inhibitor	N-Methylnicotinamide	Nicotinamide N-methyltransferase (NNMT)	NNMT inhibitors reduce SAM level and histone methylation in NNMT-overexpressed cells	NA	Kraus et al. (2014)

2-DG, 2-Deoxyglucose; GLS, glutaminase; AML, acute myeloid leukemia; ALL, acute lymphocytic leukemia; MM, multiple myeloma; NHL, Non-Hodgkin Lymphoma; NA, not applicable.

Glycolysis inhibitors could reverse global histone hyperacetylation. 2-Deoxyglucose (2-DG), a glucose analog, is a rate-limiting enzyme for glycolysis. The use of 2-DG inhibits acetyl-CoA levels, which rationally promotes histone deacetylation in multiple cancer cell lines. Hence, glycolysis inhibition represents a candidate target for regulating histone acetylation. Glutaminolysis produces α− KG and acetyl-CoA. Glutaminase (GLS) is an extensively investigated target. Relevant inhibitors include CB-839, compound 968, and BPTEs. For example, compound-968 suppresses histone H3K4me3 in breast cancer and Zaprinast decreases H3K9Me3 in IDH-mutant cancer cells. The utility of GLS inhibitors could restore epigenetic dysfunction, particularly in IDH 1/2-mutant tumors. In addition, IDH 1/2 inhibitors specifically reduce the production of 2-HG that is an oncometabolite in IDH 1/2-mutant cells. For instance, AG-221 and AGI-6780 treatment result in demethylation status of DNA and histone in IDH 2-mutant tumors; AGI-5198 prompts demethylation of H3K9me3 and H3K27me3 in chondrosarcoma cells; GSK-321 causes DNA hypomethylation in AML cells. NNMT inhibitors lead to reduced SAM levels, which in turn downregulate histone methylation. The summarized concepts are illustrated in Table 4.

### 4.3 Reversal of metabolism rewiring by targeting epigenetics

Instead, using epigenetic drugs could modulate metabolism rewiring as well (Table 5).

**TABLE 5:** Reversal of metabolism reprogramming by targeting epigenetics.

Inhibitors	Target enzyme	Pharmacological agents	Mechanism	Indication	References
DNMT inhibitor	DNA methyltransferases	Azacitidine (approved)	Non-selective inactivating DNMT1, DNMT3A, and DNMT3B; Reversing the hypermethylation status in IDH1-mutant glioma cells	MDS, AML	Borodovsky et al. (2013); Turcan et al. (2013)
		Decitabine (approved)			
		Guadecitabine (phase-III)			
KDM inhibitor	LSD1 (Lysine demethylase)	ORY-1001 (phase-I)	Inhibiting histone demethylation	AML, SCLC, MDS	NCT02913443
		GSK2879552 (phase-I)			NCT02177812
					NCT02034123
HDAC inhibitor	Histone deacetylases	Romidepsin (approved)	Prompting histone acetylation; Reducing glucose uptake, glycolytic flux, and lactate metabolism	T-cell Lymphoma, MM	Wardell et al. (2009); Alcarraz-Vizán et al. (2010); Amoêdo et al. (2011); Rodrigues et al. (2015)
		Vorinostat (approved)			
		Panobinstat (approved)			
		Belinostat (approved)			
SIRT activator and inhibitor	SIRT6 (Histone deacetylases)	Linoleic acid	Activating or inhibiting histone deacetylation; Free fatty acid activates SIRT6 that inhibits glycolysis	Unknown	Feldman et al. (2013)
		Myristic acid			
		Oleic acid			
miRNA modulator	miRNAs	miRNA mimics	miRNA reversed silenced miRNA function; miRNA-143 could inhibit glycolysis by targeting hexokinase-II 3′-UTR; Anti-miRNA-21 could restore PTEN expression	Unknown	Meng et al. (2007); Gregersen et al. (2012)
		miRNA sponges			
		antisense oligonucleotides			

DNMT, DNA, methyltransferase; KDM, lysine demethylase; HDAC, histone deacetylase; SIRT, sirtuin; miRNA, microRNA; MDS, myelodysplastic syndrome; AML, acute myeloid leukemia; SCLC, small cell lung cancer; MM, multiple myeloma; 3′-UTR, 3′-untranslated region.

There are two kinds of DNMT inhibitors therapeutically targeting DNA methylation, respectively named 5-azacytidine and 5-aza-2′-deoxycytidine. Both of them have been approved by FDA to treat myelodysplastic syndrome (MDS). IDH 1/2-mutant tumors carrying DNA hypermethylation show a high sensitivity to DNMT inhibitor. In IDH 1-mutant glioma models, both of 5-azacytidine and 5-aza-2′-deoxycytidine induced tumor regression. When inducing the differentiation of IDH-mutant glioma cells, 5-aza-2′-deoxycytidine displayed a more potent efficacy than IDH inhibitors. Therefore, targeting epigenetics is a complementary approach to modulate the effect of oncometabolites in tumor. HDAC inhibitors could induce histone acetylation and reverse gene silence caused by HDACs. Growing evidence suggests HDAC inhibitors significantly suppressed glycolysis in various cancer types, such as lung cancer, breast cancer, and multiple myeloma. These findings manifest that inhibition of HDAC might reverse glycolytic phenotype. The modulation of SIRT activator and inhibitor holds promise as their regulatory roles in metabolism reprogramming. MiRNA-based therapeutics, such as miRNA-143, also inhibit glycolysis by targeting hexokinase-II 3′-UTR. More examples are summarized in Table 5.

### 4.4 Combination therapy of metabolism and epigenetics

Advancements in the area of cancer drug discovery have spotlighted on the inhibitors of metabolic pathways and cancer epigenetics. However, the efficacy of epigenetic inhibitors alone is not satisfactory, and this approach is usually prone to drug resistance (Zhang et al., 2020). Also, cancer cell could be drug-resistant to suppression of a particular metabolic pathway by upregulating compensatory pathways or expressing alternative isoforms. Further, inhibitions of metabolic enzymes might produce systemic toxicity owing to their physiological role in normal cells (Pearce et al., 2013; Ito and Suda, 2014; Erez and DeBerardinis, 2015). To achieve the purpose of less toxicity and potent efficiency, a rational strategy is to develop multiple drug combinations.

As an epigenetic regulator, enhancer of zeste homology (EZH2) inhibits gene transcription by trimethylation of histone H3K27 in cancer cells. Mounting evidence has suggested that EZH2 participated in the alteration of metabolic profiles in cancer through diverse pathways, covering glucose, lipid, amino acid metabolism. Meanwhile, metabolic activities also affect the stability and methyltransferase activity of EZH2, as some metabolites offer the donors for EZH2 post-translational modifications (Zhang et al., 2020). As a promising target, EZH2 inhibitors have been investigated in preclinical trials, but the effectiveness of EZH2 inhibitors alone is not satisfactory (De Raedt et al., 2011; Baude et al., 2014; Huang X. et al., 2018). Recently, researchers have found EZH2 inhibitor is able to weaken drug resistance caused by metabolic activities in tumor. Solid tumor is subject to hypoxia and glutamine deficiency because of the underdeveloped vascular system. Hypoxia induces a metabolic switch from oxidative to glycolytic metabolism, promoting the dedifferentiation of tumor cells and inducing resistance to radio- and chemotherapy. However, EZH2 inhibitors could directly block H3K27 methylation and consequently activate the transcription of pro-differentiation genes. Also, metabolic pathway is likely to downregulate EZH2 activity and thereby acts synergistically with EZH2 inhibitors (Zhang et al., 2020). More specifically, AMPK is activated in response to energy stress (glucose deficiency) and phosphorylates EZH2 (Cha et al., 2005). AKT-mediated phosphorylation of EZH2 suppresses trimethylation of lysine 27 in histone H3, facilitating the transcription of target genes to suppress tumor growth (Cha et al., 2005; Priebe et al., 2011; Gao et al., 2014; Kim and Yeom, 2018). Therefore, a combination of EZH2 inhibitors with metabolic regulators is a novel strategy to rescue the poor effectiveness of EZH2 inhibitor alone (Zhang et al., 2020). Briefly, epigenetic and metabolic alterations mediated by EZH2 are highly interlaced, demonstrating a synergistic effect in treating malignancy.

A model whereby linked metabolic-epigenetic programs reflects a new idea to target such an integrated axis. A study (McDonald et al., 2017) on the evolution of pancreatic ductal adenocarcinoma (PDAC) introduced an epigenetic mechanism that links glucose metabolism to distant metastasis. Remarkably, oxidative branch of the Pentose Phosphate Pathway (ox-PPP) was a driving force for epigenetic programming (histone H3K9 and DNA methylation) that enhanced tumorigenic fitness during the distant metastasis. Hence, targeting ox-PPP to reverse malignant epigenetic programs could be effective in metastatic PDAC. Another best-studied example is the use of AMPK activator metformin, which decreased EZHIP protein concentrations, elevated H3K27me3, inhibited TCA cycle, and suppressed tumor growth. Consequently, targeting integrated epigenetic-metabolic pathway shows hopeful therapeutic efficacy in mice models transplanted with PFA ependymomas (Panwalkar et al., 2021).

Oncogenic signal pathways also play important roles in novel combination therapy. A distinct work on melanoma demonstrated that reduced α -KG levels result in histone hypermethylation and develop the resistance to BRAF inhibitors. The combination of histone methyltransferase and BRAF inhibitors was sufficient to overcome resistance (Pan et al., 2016). Also, liver kinase B1 (LKB1)-deficiency tumors carrying KRAS activation would accompany with SAM production, leading to more potent methyltransferase activity and increased DNA methylation levels (Kottakis et al., 2016). Combined inhibition of DNA methyltransferase and serine metabolism could attack LKB-loss tumors with KRAS-positive more aggressively.

Taken together, our understanding in targeting both altered metabolism and epigenetics remains at a very early stage. Whether these two hallmarks exert synergistic functions in tumor is less explored, though there are a few well-elaborated agents in ongoing clinical trials (Table 6).

**TABLE 6:** Ongoing clinical trials of combined anti-epigenetic drugs and anti-metabolism drugs.

Identifier	Start year	Combination therapy		Conditions	Phase	Enrollment
		Anti-epigenetics drug	Anti-metabolism drug			
NCT02719574	2016	Azacitidine	FT-2102	AML/MDS	I/II	336
NCT02677922	2016	Azacitidine	AG-120	AML	I/II	131
NCT03173248	2017	Azacitidine	AG-120	AML	III	148
NCT03471260	2018	Azacitidine	AG-120	Hematologic malignancies	I/II	30
NCT03683433	2018	Azacitidine	AG-221	AML	II	50
NCT03684811	2018	Azacitidine	FT-2102	Solid tumors and gliomas	I/II	200
NCT04774393	2021	Decitabine	AG-120/AG-221	AML	I/II	84

AML, acute myeloid leukemia; MDS, myelodysplastic syndrome; DNMT, inhibitors: Azacitidine; Decitabine. IDH, inhibitors: AG-120 (Ivosidenib); AG-221 (Enasidenib); FT-2102.

## 5 Epigenetic, metabolic, and immune crosstalk

### 5.1 Principles linking cancer metabolism, epigenetics, and immunity

In the traditional viewpoint, immunological memory is a unique feature of the adaptive immune system (Netea et al., 2020a). However, “Trained immunity” is a relatively new term that refers to myeloid cells from the innate immune system also display memory capacity after pathogen exposure (Dominguez-Andres and Netea, 2019; Netea et al., 2020b; O'Neill and Netea, 2020). After the first stimuli, innate immune cells, such as macrophage and monocyte, are epigenetically programmed (Fanucchi et al., 2021). These epigenetic modifications unfold chromatin and expose promoter and enhancer regions controlling immune-associated genes, enabling them accessible to transcription factors (Klemm et al., 2019) and permitting cells to maintain a “trained” state after rechallenge (Saeed et al., 2014). Specifically, H3K4me3 frequently occurs on gene promoters; H3K4me1 and H3K27Ac accumulates on enhancers (Quintin et al., 2012; Novakovic et al., 2016). As such, upon the secondary stimulus, immune genes are more robustly transcribed (Fanucchi et al., 2021).

In addition, some metabolites act as substrates or co-factors for epigenetic enzymes, which alter chromatin state to cause transcriptional changes that are causal to trained immunity (Fanucchi et al., 2021). For example, acetyl-CoA mediates histone acetylation following immune stimuli (Wellen et al., 2009; Christ and Latz, 2019), while SAM level regulates DNA and histone methylation to control trained immunity (Mentch et al., 2015; Ji et al., 2019). On the contrary, NAD^+^ assist histone deacetylation to block trained immunity (Yeung et al., 2004; Zhong et al., 2010; Lo Sasso et al., 2014; Jia et al., 2018). α -KG-derived metabolites reduce histone demethylation by competing with α -KG-dependent KDM5 histone demethylase (Sowter et al., 2003; Cheng et al., 2014). Explicitly, human monocytes exposed to β -glucan will have higher concentrations of α -KG-derived metabolites and lower activity of KDM5 demethylases, which is associated with less H3K4me3 demethylation and higher gene expression (Fanucchi et al., 2021). Overall, the induction, maintenance, and regulation of “trained immunity” is based on the complex interplay between epigenetics and metabolism.

Apart from trained immunity, the crosstalk of metabolism and epigenetics has been reported in T cell (Bailis et al., 2019) and macrophage activation (Liu et al., 2017). A recent study has shown that both mitochondrial citrate export and malate-aspartate shuttle favor histone acetylation and influence the expression of specific genes involved in T cell activation (Bailis et al., 2019). Also, a research figured out α -KG produced from glutamine metabolism orchestrates M2 macrophage activation by Jmjd3-dependent epigenetic remodeling (Liu et al., 2017). Specifically, H3K27me3 is a repressive epigenetic marker that downregulates the expression of M2 macrophage marker genes (Ishii et al., 2009). It is notable Jmjd3 is a crucial enzyme for demethylation of H3K27 (Satoh et al., 2010). α -KG derived from glutamine metabolism could facilitate epigenetic changes in a Jmjd3-dependent demethylation of H3K27 on the promoters of M2-specific marker genes (Bailis et al., 2019). This result indicates α -KG and Jmjd3 synergistically promotes macrophage activation. Consequently, an attractive strategy is to modulate glutamine metabolism to harness macrophage-mediated immune responses.

### 5.2 Rational for novel immunotherapy-based combinations

Cancer immunotherapy is rapidly developing in various research settings, including CAR-T cell therapy, immune checkpoint inhibitors, and adoptive transfer of tumor infiltrating lymphocytes (Rosenberg et al., 1988; Zhao et al., 2005; Robbins et al., 2011; Rosenberg et al., 2011; Rosenberg, 2012; Topalian et al., 2012; Maude et al., 2014). An innovative strategy is the combination of immunotherapy with either epigenetic inhibitors or metabolic inhibitors, or a triple combination of them.

Epigenetics and immunology are both fast-developing fields in cancer biology. Recent evidence provides unique opportunities to combine epigenetics-based drugs with immunotherapy (Zhang et al., 2020). Epigenetic-based drugs include four pan-HDAC inhibitors and two DNMT inhibitors approved by FDA before 2020 (Knutson et al., 2012; Yu et al., 2017). These agents are able to change the immunosuppressive tumor microenvironment and increased tumor-infiltrating lymphocytes (Yanagida et al., 2001; Wang L. et al., 2010; Li et al., 2013; Anwar et al., 2018), leading to enhanced tumor-associated antigen presentation, activation of DC cells, suppression of T cell exhaustion. Similar changes in TME are also observed in tumor tissues treated with other agents, such as inhibitors of KMT6A (EZH2), KDM1A (LSD1), PRMT5, and BET proteins (Hemmings and Restuccia, 2012; Kikuchi et al., 2015; Garcia and Shaw, 2017; Herzig and Shaw, 2018; Hoxhaj and Manning, 2020). Consequently, given that epigenetic drugs boosting antitumor immune response, immune checkpoint blockade therapies (ICBTs) and epigenetic-based inhibitors exert synergistic functions to sensitize less-immunogenic tumors and prevent both primary and acquired resistance (Zhang et al., 2020). There are numerous ongoing clinical trials summarized in Table 7.

**TABLE 7:** Ongoing clinical trials of combined anti-epigenetic drugs and immune checkpoint inhibitors.

Identifier	Start year	Combination therapy		Conditions	Phase	Enrollment
		DNMT inhibitors	Checkpoint inhibitor			
NCT02608437	2015	Guadecitabine	Ipilimumab	Metastatic melanoma	I	19
NCT02530463	2015	Azacitidine	Ipilimumab/Nivolumab	MDS/Leukemia	II	160
NCT02957968	2016	Decitabine	Pembrolizumab	Breast cancer	II	32
NCT02890329	2016	Decitabine	Ipilimumab	MDS/AML	I	48
NCT02664181	2017	Decitabine	Nivolumab	NSCLC	II	13
NCT03094637	2017	Azacitidine	Pembrolizumab	High-risk MDS	II	37
NCT03264404	2017	Azacitidine	Pembrolizumab	Pancreas cancer	II	31
NCT03019003	2017	Azacitidine	Durvalumab	Head and neck cancer	I/II	13
NCT03308396	2017	Guadecitabine	Durvalumab	Kidney cancer	Ib/II	57
NCT04510610	2019	Decitabine	Camrelizumab	Hodgkin lymphoma	II/III	100
NCT04353479	2020	Decitabine	Camrelizumab	AML	II	29

**Identifier**	**Start Year**	**Combination Therapy**		**Conditions**	**Phase**	**Enrollment**
		**HDAC Inhibitors**	**Checkpoint Inhibitor**			
NCT02616965	2015	Romidepsin	Brentuximab vedotin	T-cell lymphoma	I	27
NCT03024437	2017	Entinostat	Atezolizumab	Renal cancer	I/II	72
NCT03848754	2019	Pracinostat	Gemtuzumab ozogamicin	AML	I	14
NCT03903458	2019	Tinostamustine	Nivolumab	Advanced melanoma	IB	21
NCT03820596	2019	Chidamide	Sintilimab	NK/T-cell lymphoma	I/II	50
NCT04651127	2020	Chidamide	Toripalimab	Cervical cancer	I/II	40
NCT04562311	2020	Chidamide	Tislelizumab	Bladder cancer	II	43

**Identifier**	**Start Year**	**Combination Therapy**		**Conditions**	**Phase**	**Enrollment**
		**KMT6A Inhibitor**	**Checkpoint Inhibitor**			
NCT03525795	2018	CPI-1205	Ipilimumab	Advanced solid tumor	I	24
NCT03854474	2019	Tazemetostat	Pembrolizumab	Bladder cancer	I/II	30

**Identifier**	**Start Year**	**Combination Therapy**		**Conditions**	**Phase**	**Enrollment**
		**KDM1A inhibitor**	**Checkpoint Inhibitor**			
NCT02712905	2016	INCB059872	Nivolumab	Hematologic tumor	I/II	116
NCT02959437	2017	INCB059872	Pembrolizumab	Hematologic tumor	I/II	70

MDS, myelodysplastic syndrome; AML, acute myeloid leukemia; NSCLC, non-small cell lung cancer.

Metabolism can be modulated *in vivo* to govern anti-tumor T cell longevity and functionality, which determines the efficacy of immunotherapy (Chang and Pearce, 2016; O'Neill et al., 2016). The modulation of T cell metabolism is a promising strategy to enhance or suppress immune response (O'Sullivan and Pearce, 2015), as the characteristics of T cells are critical to determine clinical outcomes (Klebanoff et al., 2012). Several advances have been made in preclinical models. For example, when treating vascularized melanoma, limiting the ability of T cells engaged in glycolysis through suppression of hexokinase by 2-DG could ultimately leads to enhanced anti-tumor efficacy (Sukumar et al., 2013). Additionally, metabolic reprogramming occurs in other immune cells within tumor microenvironment, such as macrophages and dendritic cells (DCs). One research (Yan et al., 2021) put forward strategies to enhance cancer immunotherapy by manipulating metabolism reprogramming. For example, CB-839 is a glutaminase inhibitor that has been explored in numerous clinical trials with or without the combinations of immunotherapy (Cerezo and Rocchi, 2020). Acetyl-CoA acetyltransferase 1 (ACAT1) inhibitors could enhance the activity of CD8^+^ T cells and reduce the inflammatory response. Hence, ACAT1 might be a potential target to optimize immunotherapy (Yang et al., 2016; Huang L. H. et al., 2018; Bi et al., 2019). Indoleamine 2,3-dioxygenase (IDO) is responsible for the conversion of tryptophan to kynurenine in tumors. Blocking IDO can decrease Treg cells and preserve the functionality of T cells. Combination of IDO inhibitors (epacadostat) and immune checkpoint inhibitor (pembrolizumab) has been shown safe enough in clinical trials, though its efficacy needs further investigation (Prendergast et al., 2017; Komiya and Huang, 2018; Long et al., 2019). In summary, glutamine, acetyl-CoA acetyltransferase 1 (ATAC1), indoleamine 2,3-dioxygenase (IDO), lactate, and Toll-like receptors (TLRs) are likely to be considered as novel “metabolic checkpoints”, targeting of which could assist immune cells to achieve better anti-tumor effect.

Noteworthily, epigenetic, metabolism, and immune crosslink in germinal-cancer-derived B-cell lymphomas (GCB) uncover a rational triple combination therapy (Serganova et al., 2021). GCB lymphoma is significantly heterogenous based on genetic, epigenetic, and clinical characteristics. Epigenetic dysfunction, such as gain-of-function mutations of EZH2 and loss-of-function mutations of CREBP and EP300, disrupts the normal biological link between lymphoma cells and immune TME, and motivates immune evasion in GCB lymphoma. Also, lymphoma metabolism adaptions might aggravate immunosuppression, leading to poorly infiltrated effector T-cell. Considering the impacts of cancer metabolism on epigenetic modifier and immune microenvironment, triple combination therapy is a logic and feasible strategy for future treatment.

## 6 Perspectives

**FIGURE 3:**
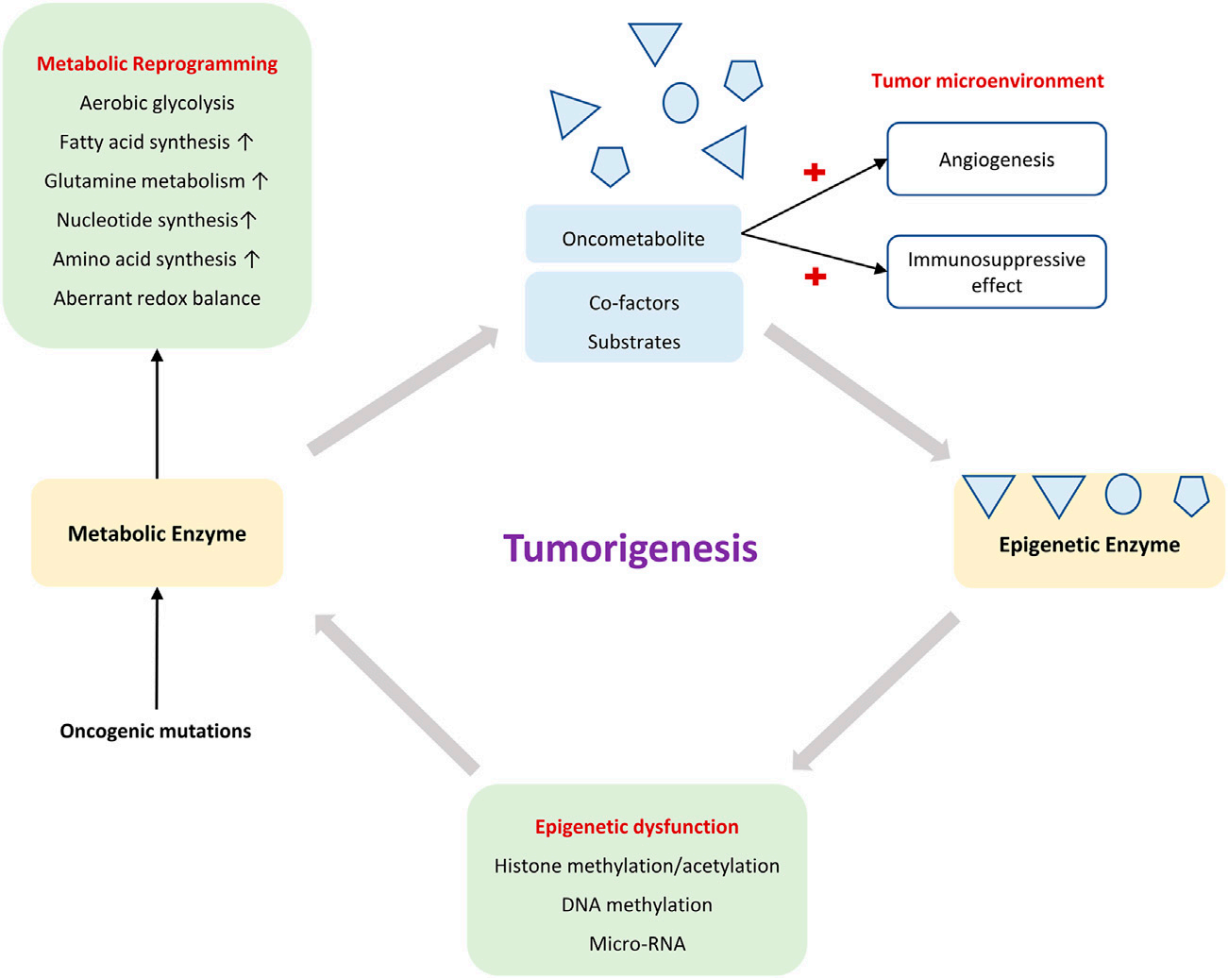
The crosstalk between metabolism and epigenetics in tumorigenesis.

As reviewed, epigenetics and metabolism are highly interconnected in a reciprocal manner (Figure 3 **)**. Such a relationship is accentuated by the reversibility of both processes (Henikoff and Matzke, 1997). A major goal in exploring metabolism-dependent epigenetic modifications is the hope of identifying novel targets for cancer therapy. However, some aspects pertaining to metabolic-epigenetic axis in cancers remain poorly understood.

Firstly, tumor heterogeneity is a major challenge that limits our understanding (Hensley et al., 2016). Inconsistent metabolic phenotypes were observed in various tumor tissues. Hence, tumor heterogeneity allows cancer cells to escape the deleterious attacks of inhibitors (Thakur and Chen, 2019). Secondly, the downstream factors mediating the tumorigenic activity of oncometabolites remains largely unknown. Thirdly, enzymatic parameters, such as K_m_, V_max_, and allosteric and inhibitory binding constants, constitute the basic element of the biochemistry (Reid et al., 2017). It is difficult to define physiological conditions in which the concentration dynamics of substrates and co-factors causally underlie an alteration of chromatin status. Discrepancies exist between artificial culture *in vitro* and physiological environment *in vivo* (Davidson et al., 2016). Another complexity is the precise input of metabolism into chromatin modifications, as both activation and suppression of histone marks need metabolites. For instance, how to predict the changes of SAM level establish the overall chromatin state and epigenetic phenotype. Additionally, though a bunch of metabolic enzymes function in nucleus have been identified, their individual contribution to epigenetic alterations was less defined. Robust experimental methods are needed to obtain accurate measurements of metabolites in specific cellular domain. Despite much interest in targeting both metabolism and epigenetics, poorly understood layers that whether these two hallmarks confer dependencies in tumors synergistically still exist.

In-depth connection between oncogenic signaling, metabolism, epigenetics, and immunity in cancer would facilitates effective designing of novel targeted drugs, which is the premise of precision medicine. It is anticipated that multiple combination therapies hold opportunities to improve care of cancer patients. Nevertheless, several outstanding challenges will be the major goal of future study.
